# Deciphering the Relationship Between Alcohol Consumption, Oropharyngeal Cancers, and Socioeconomic Determinants

**DOI:** 10.1111/acer.70393

**Published:** 2026-08-05

**Authors:** Tareq Al‐Ahdal, Brenda Cabrera‐Mendoza, Stefan Listl, Renato Polimanti

**Affiliations:** ^1^ Heidelberg Institute of Global Health, Section for Oral Health Faculty of Medicine and University Hospital, Heidelberg University Heidelberg Germany; ^2^ Interdisciplinary Center for Scientific Computing, Heidelberg University Heidelberg Germany; ^3^ Department of Psychiatry Yale University School of Medicine New Haven Connecticut USA; ^4^ Veteran Affairs Connecticut Healthcare System West Haven Connecticut USA; ^5^ Department of Chronic Disease Epidemiology Yale University School of Public Health New Haven Connecticut USA; ^6^ Department of Biomedical Informatics & Data Science Yale University School of Medicine New Haven Connecticut USA; ^7^ Wu Tsai Institute, Yale University New Haven Connecticut USA

**Keywords:** alcohol consumption, genome‐wide association study, household income, instrumental variable analysis, oral health

## Abstract

**Background:**

Alcohol drinking is a major risk factor for oropharyngeal cancer, yet the nature of risk differences across drinking patterns remains unclear.

**Methods:**

We leveraged data from large‐scale genome‐wide association studies to conduct Mendelian randomization analyses, assessing the effects of alcohol intake with meals, intake frequency, maximum habitual intake, and drinks per week on risks of oral cavity cancer, pharyngeal cancer, stomatitis, lichen planus, and oral leucoplakia. Multiple Mendelian randomization methods were applied to model pleiotropic effects, and multivariable analysis was performed to evaluate the interplay between alcohol consumption and household income.

**Results:**

Alcohol intake frequency was associated with increased risk of oral and pharyngeal cancer. In contrast, alcohol intake with meals showed an apparent protective association with these outcomes, which was nullified after adjusting for household income in the multivariable analysis—indicating that the observed benefit was explained by higher socioeconomic status. The frequency‐risk relationship remained significant after adjustment, highlighting intake frequency as an independent risk factor under MR assumptions. The number of drinks per week was linked to increased stomatitis risk, and this association remained significant after accounting for household income, suggesting an independent effect of alcohol consumption on stomatitis risk under MR assumptions.

**Conclusion:**

Cancer prevention strategies should target harmful alcohol consumption patterns with attention to drinking frequency and socioeconomic context, rather than treating alcohol exposure as monolithic.

## Graphical abstract

Using Mendelian randomization, we show that alcohol intake frequency increases oropharyngeal cancer risk independently of socioeconomic status, while the apparent protective effect of drinking with meals is explained by higher household income. Drinks per week independently increase stomatitis risk. Cancer prevention should target specific drinking patterns alongside socioeconomic determinants.

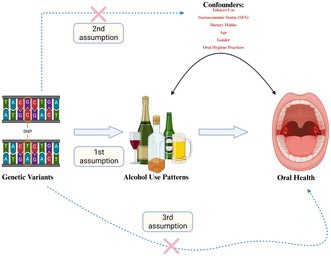

## 1 Introduction

Oral diseases affect millions of people worldwide and can cause severe functional impairment, chronic pain, and disfigurement (Peres et al. 2019). Among them, oral cancer is one of the most serious conditions of the oral cavity, with high morbidity and mortality, particularly when diagnosed at advanced stages (Omar 2015; Warnakulasuriya 2009). Beyond the immediate threat to life, these diseases can also limit the ability to speak, eat, and socialize, ultimately reducing quality of life (WHO 2024).

Alcohol drinking is one of the leading risk factors in the global disease burden (Lim et al. 2012). In particular, observational studies and meta‐analyses highlighted its impact on different types of oral cancer (Turati et al. 2013) and other oral health outcomes (Kusu et al. 2025). However, while growing evidence highlights that no amount of alcohol drinking can be considered safe (Barbería‐Latasa et al. 2022), there is limited information regarding the oral‐health risk associated with different patterns of alcohol consumption. Because a large proportion of worldwide populations drink alcohol at least in small quantities (Bryazka et al. 2022), understanding the dynamics linking different alcohol drinking patterns to negative oral health outcomes has major public health implications and can contribute to defining evidence‐based recommendations. To our knowledge, no study has systematically investigated alcohol drinking patterns with respect to different types of oral cancer and other oral health outcomes.

Mendelian randomization (MR) is a powerful design to rapidly gain insights regarding possible direct effects between exposures and outcomes of interest, leveraging genetic information as instrumental variables (Sanderson et al. 2022). Data from large‐scale genome‐wide association studies (GWAS) of different alcohol drinking patterns and oral health outcomes are available from large biobanks and international consortia, including UK Biobank (UKB) (Bycroft et al. 2018), Million Veteran Program (MVP) (Gaziano et al. 2016), FinnGen Project (Kurki et al. 2023), the GWAS & Sequencing Consortium of Alcohol and Nicotine use (GSCAN) (Saunders et al. 2022), and the International Head and Neck Cancer Epidemiology (INHANCE) Consortium (Lesseur et al. 2016). To account for possible confounders affecting our findings, we used multiple MR approaches to assess the effect of alcohol drinking patterns on oral cancer and other oral health outcomes. Additionally, because there is a complex interplay between alcohol consumption, socioeconomic factors, and human health (Collins 2016; Probst et al. 2020), we performed a multivariable MR analysis to model the direct effect of alcohol drinking patterns on oral health, accounting for indirect relationships related to household income.

## 2 Methods

### 2.1 Study Design

The study was designed in accordance with the recommendations of the Strengthening the Reporting of Observational Studies in Epidemiology using Mendelian Randomization (STROBE‐MR) report (Skrivankova et al. 2021) (the related checklist is included in the Supporting Information).

### 2.2 Ethics Statement

Because the study was conducted using previously generated, de‐identified, aggregated data, ethical approval was not required. Details regarding the ethical approval of the studies that generated these datasets can be found elsewhere (Bycroft et al. 2018; Gaziano et al. 2016; Kurki et al. 2023; Lesseur et al. 2016; Saunders et al. 2022).

### 2.3 Data Sources

Information regarding exposures and outcomes was derived from previous GWAS. Specifically, we analyzed genome‐wide data related to four alcohol drinking phenotypes. GWAS of alcohol intake frequency and alcohol usually taken with meals were obtained from MRC IEU OpenGWAS (dataset ID: ukb‐b‐5779 and ukb‐b‐16878, respectively) (Elsworth et al. 2020). Both were based on UKB participants of European descent (EUR). The first was performed in 462,346 participants assessed through a six‐category scale ranging from never to daily or almost daily. As the phenotype was standardized prior to GWAS, effect estimates represent changes per one standard deviation increase in alcohol intake frequency. (UKB Data‐Field 1558). The GWAS of alcohol usually taken with meals was based on a binary definition, including 159,104 cases and 76,541 controls. The exposure represents the odds of usually drinking alcohol with meals compared to not (UKB Data‐Field 1618). From MVP, we derived an additional alcohol drinking phenotype, maximum habitual alcohol intake in a day (Deak et al. 2022). This was assessed in 218,623 individuals using the following survey item: “In a typical month, what is/was the largest number of drinks of alcohol you may have had in one day?” with ordinal responses from 0 to 15 or more drinks. As the phenotype was standardized prior to GWAS, effect estimates represent changes per one standard deviation increase in maximum habitual alcohol intake. Finally, we derived genome‐wide information regarding the number of alcoholic drinks per week from the publicly available GSCAN dataset (Saunders et al. 2022), including up to 666,978 EUR participants. As the phenotype was standardized prior to GWAS, effect estimates represent changes per one standard deviation increase in drinks per week. A total of six oral health outcomes were investigated. These included three cancer‐related phenotypes available from INHANCE GWAS meta‐analysis (Lesseur et al. 2016): oral cavity cancer (dataset ID: ieu‐b‐94; 1223 cases, 2928 controls), pharyngeal cancer (dataset ID: ieu‐b‐96; 1090 cases, 2928 controls), and oral cavity and pharyngeal cancer (dataset ID: ieu‐b‐89; 2497 cases, 2928 controls). Three non‐cancer oral health phenotypes were derived from the FinnGen GWAS Release 12: stomatitis (FinnGen endpoint: K11_STOMATITIS; 3437 cases, 310,260 controls), lichen planus (FinnGen endpoint: K11_LICHEN_PLANUS_WIDE; 8507 cases, 491,841 controls), and oral leukoplakia (FinnGen endpoint: K11_ORAL_LEUCOPLAC_RELAT_INCLAVO; 596 cases, 499,752 controls). Details regarding FinnGen GWAS are available at https://finngen.gitbook.io/documentation/methods/phewas.

To assess whether the relationship between alcohol drinking phenotypes and oral health is at least partially influenced by socioeconomic factors, we used genome‐wide information related to household income available from a GWAS meta‐analysis with an effective sample size of 471,573 (Kweon et al. 2025).

Full details of genotyping platforms, quality control procedures, and principal components used are described in the original publications for each dataset.

### 2.4 Mendelian Randomization

From GWAS of alcohol drinking phenotypes, we selected genome‐wide significant variants (*p* < 5 × 10^−8^) and clumped them to define independent genetic instruments (linkage disequilibrium, LD *r* ^2^ < 0.01, window size 10,000 kb) based on the 1000 Genomes Project Phase 3 reference EUR populations (Consortium 2015). Palindromic SNPs are excluded to avoid allele misalignment between exposure and outcome datasets. To reduce potential weak instrument bias, we limited our analyses to exposures with genetic instruments with an average *F*‐statistic > 10. The number of genetic instruments varied across exposure‐outcome combinations due to SNP attrition during harmonization. Specifically, after clumping, the number of instruments retained after harmonization ranged from 7 to 88 SNPs depending on the exposure‐outcome pair, with cancer outcomes (INHANCE) retaining fewer SNPs than FinnGen outcomes due to differences in genome‐wide coverage between datasets. *F*‐statistics for each exposure‐outcome analysis are reported in the Tables S1–S9.

We estimated the effect of alcohol drinking patterns on oral health outcomes using a two‐sample MR framework (Sanderson et al. 2022). We used multiple MR methods to account for different pleiotropy dynamics affecting instrument validity. While it has higher statistical power, the inverse‐variance weighted (IVW) approach assumes no directional pleiotropy (Sanderson et al. 2022). Instead, MR‐Egger allows for directional pleiotropy by including an intercept term that measures the presence of directional pleiotropy (Burgess and Thompson 2017). The weighted median provides a consistent estimate if at least 50% of instruments are valid (Bowden et al. 2016). The mode‐based estimator uses the mode of the SNP‐specific causal effect distribution to be less sensitive to outliers (Burgess et al. 2018). We also performed multiple sensitivity analyses. IVW and MR‐Egger heterogeneity tests were performed using Cochran's *Q* statistic (Burgess et al. 2017). Directional pleiotropy was assessed considering the MR‐Egger regression intercept (Burgess and Thompson 2017). These MR analyses and related sensitivity analyses were conducted using the Two SampleMR R package (Hemani et al. 2018). We also used MR‐RAPS (Robust Adjusted Profile Score) to account for weak instruments and systematic pleiotropy (Score 2018), the MR‐PRESSO (Pleiotropy Residual Sum and Outlier) test to remove possible outliers (Verbanck et al. 2018), and MRlap to account for biases related to sample overlap, winner's curse, and weak instruments (Mounier and Kutalik 2023). The GSCAN drinks per week GWAS includes UK Biobank participants, creating potential sample overlap with the UK Biobank‐derived exposure GWASs. MRlap was applied to estimate and correct for this overlap. The corrected estimates were consistently slightly larger in magnitude than the observed estimates, though the direction and significance of all findings remained unchanged after correction, indicating that sample overlap had minimal impact on the primary results.

The validity of MR estimates relies on three core instrumental variable assumptions: (1) relevance genetic variants are robustly associated with the exposure (assessed using *F*‐statistics, *F* > 10 for all primary univariable analyses; conditional *F*‐statistics in the expanded multivariable models were below this threshold for smoking, risk taking, and household income, reflecting correlation among exposures); (2) independence genetic variants are independent of confounders (evaluated using MR‐Egger regression and MR‐PRESSO); and (3) exclusion restriction genetic variants affect the outcome only through the exposure (assessed using MR‐Egger intercept, weighted median, and mode‐based estimators). To follow up significant findings from univariable MR analyses, we performed multivariable MR (Sanderson et al. 2019), testing whether the effect of alcohol drinking phenotypes on oral health outcomes is independent of household income. This analysis was conducted using MVMR R package (Sanderson et al. 2021). Genetic instruments for alcohol‐related exposures and household income were harmonized across datasets, considering a genome‐wide significance threshold (***p* < 5 × 10** ^**−8**^) and the same clumping parameters used in the univariable MR analyses (LD *r* ^2^ < 0.01, 10,000‐kb window). To further explore whether the effect of alcohol intake frequency on oropharyngeal cancer is independent of other behavioral risk factors, we conducted additional multivariable MR analyses including cigarettes per day (OpenGWAS ID: ieu‐b‐25; *N* = 337,334) (Liu et al. 2019) and general risk tolerance (OpenGWAS ID: ukb‐b‐14,147) (Karlsson Linnér et al. 2019) as additional exposures alongside household income. MR‐CAUSE was additionally applied to evaluate correlated and uncorrelated pleiotropy for the primary significant associations between alcohol intake frequency and cancer outcomes, using developer‐recommended parameters (*p* < 0.001, *r* ^2^ = 0.01, clump_kb = 1000) (Morrison et al. 2020).

To avoid inference based only on *p* values, the direction and strength of effects estimated by multiple MR methods are presented in the context of their statistical significance (Sterne and Smith 2001). Given the exploratory nature of several analyses and the hypothesis‐driven focus on alcohol drinking patterns and oral health outcomes, we did not apply a formal family‐wise error rate correction across all exposure‐outcome combinations. Instead, we applied a conservative significance threshold of *p* < 0.025 for the primary MVMR analyses and *p* < 0.05 for the univariable analyses, and findings are interpreted in the context of consistency across multiple MR methods rather than relying solely on *p*‐values. Findings that were significant in only one method or at borderline thresholds are considered exploratory and should be interpreted with caution.

### 2.5 Role of the Funding Source

This research received no specific grant from any funding agency in the public, commercial, or not‐for‐profit sectors.

## 3 Results

**FIGURE 1:**
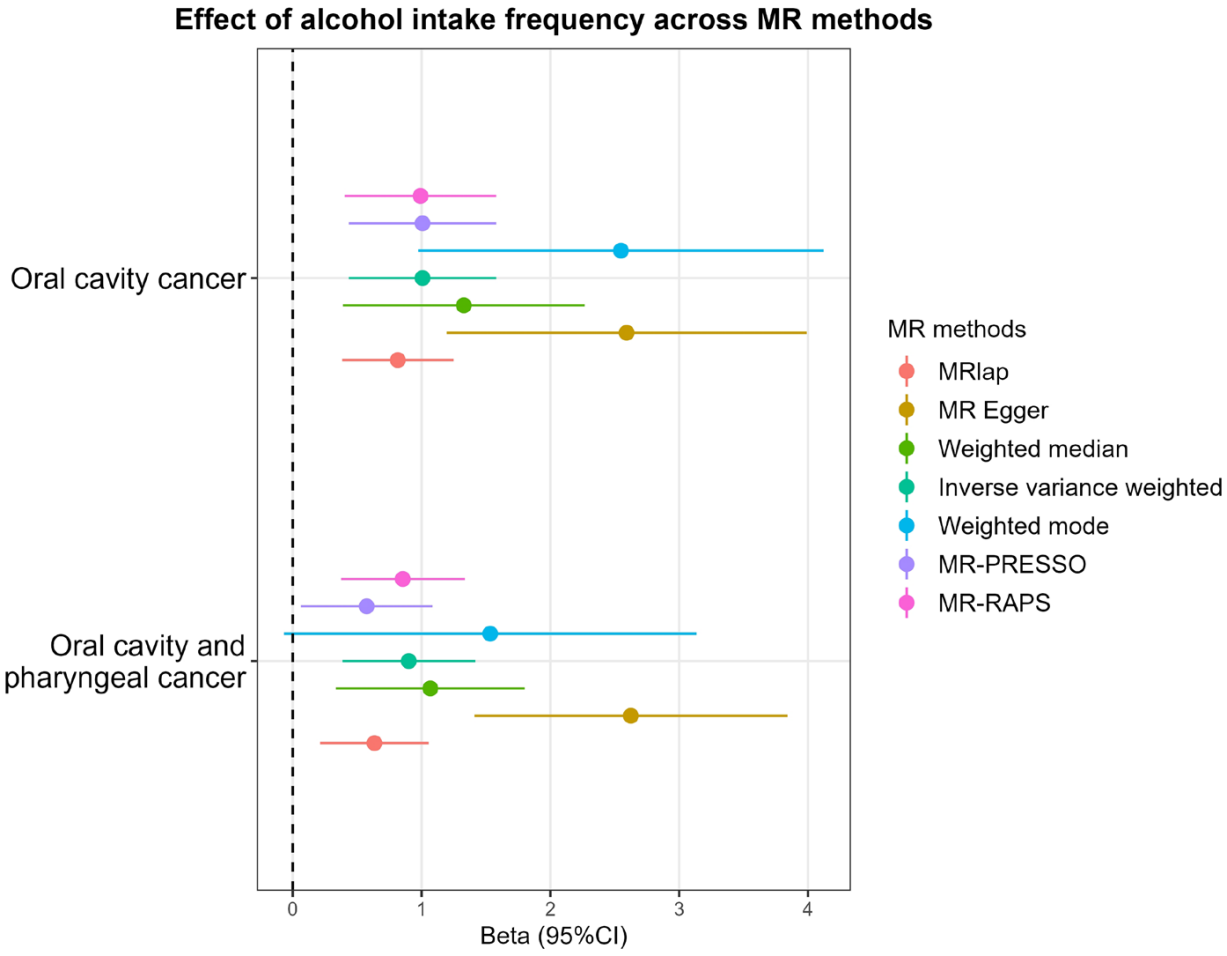
Effect of alcohol intake frequency on oral cavity cancer and oral and pharyngeal cancer across Mendelian randomization (MR) methods. Full results are available in Table S1.

**FIGURE 2:**
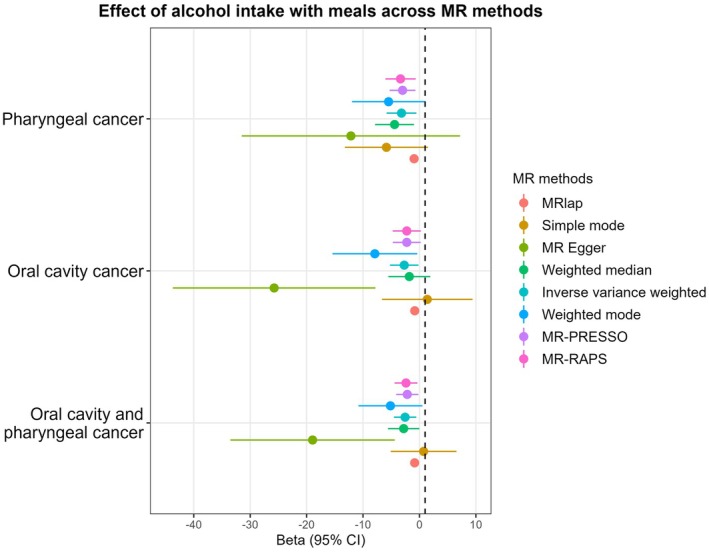
Effect of alcohol intake with meals on pharyngeal cancer, oral cavity cancer, and oral and pharyngeal cancer across Mendelian randomization (MR) methods. Full results are available in Table S2.

**FIGURE 3:**
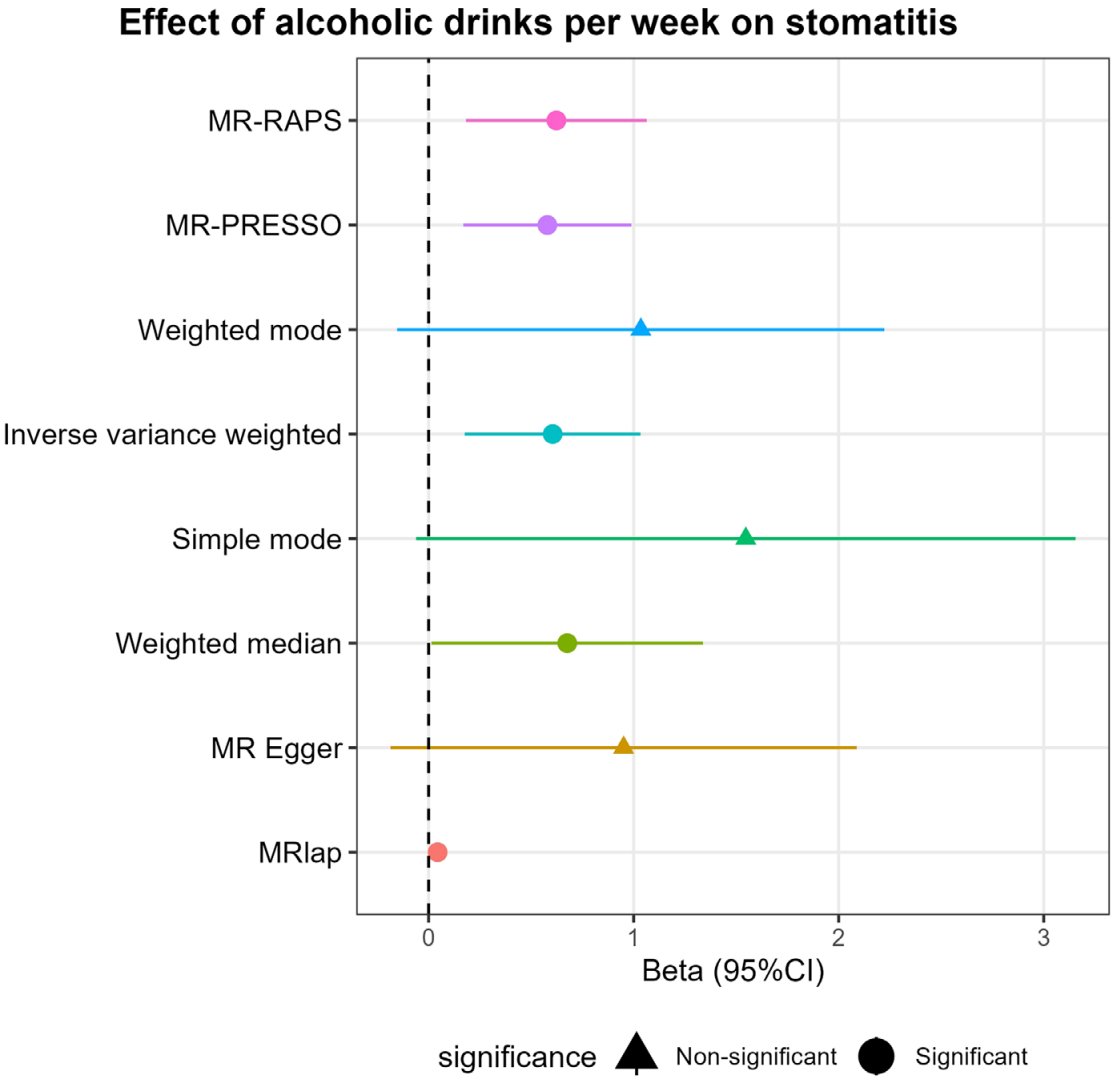
Effect of alcoholic drinks per week on stomatitis across Mendelian randomization (MR) methods. Full results are available in Table S3.

**FIGURE 4:**
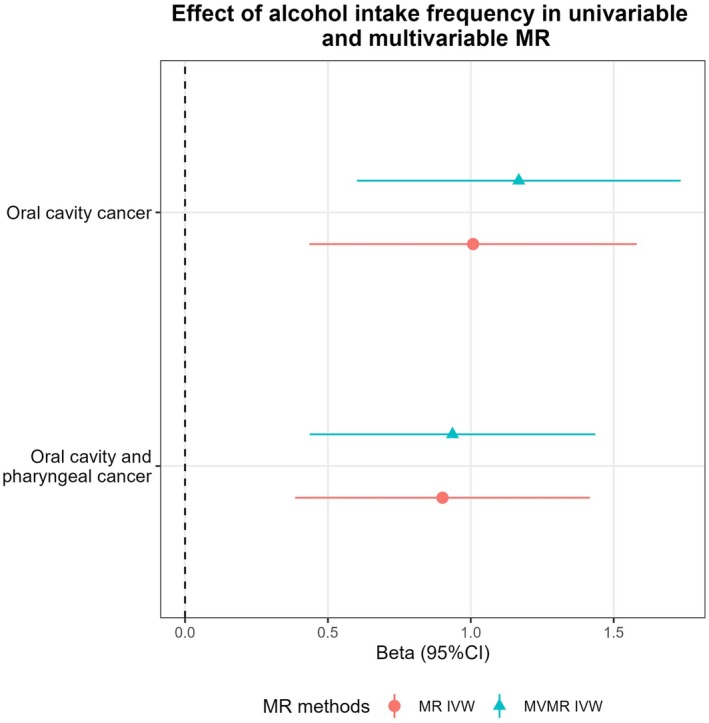
Effect of alcohol intake frequency on oral cavity cancer and oral and pharyngeal cancer considering univariable and multivariable inverse‐variance weighted approaches (MR IVW and MVMR IVW, respectively). The multivariable analysis modeled both alcohol intake frequency and household income as exposures. Full results are available in Table S5.

Our MR analyses highlighted several associations linking alcohol drinking patterns with oral cancers and other negative oral health outcomes. Alcohol intake frequency was associated with increased oral cavity cancer and oral and pharyngeal cancer across multiple MR methods (Figure 1, Table S1). The IVW estimates indicated that a one‐category increase in alcohol intake frequency was associated with a 2.74‐fold increased odds of oral cavity cancer (OR = 2.74, 95% CI: 1.55–4.86) and a 2.46‐fold increased odds of oral and pharyngeal cancer (OR = 2.46, 95% CI: 1.47–4.12). For both, there was some variation in the effect sizes estimated, with the largest one being the MR‐Egger estimate (oral cavity cancer *β* = 2.59 ± 0.71; oral and pharyngeal cancer *β* = 2.62 ± 0.62), which also indicated confirmed directional pleiotropy (MR‐Egger intercept *p* < 0.05). When pharyngeal cancer was analyzed separately, the relationship with alcohol intake frequency was significant only in the MR‐Egger analysis (*β* = 2.04 ± 0.99) without evidence of directional pleiotropy (MR‐Egger intercept *p* > 0.05), but other sensitivity analyses highlighted possible heterogeneity among the genetic instruments (Table S1). In contrast to alcohol intake frequency, alcohol intake with meals showed an inverse relationship with oral cancer, oropharyngeal cancer, and with reporting one of these cancer types (Table S2). While the effect was statistically significant across multiple MR methods, there were differences in the effect sizes (Figure 2). The IVW estimates indicated that individuals who usually drink alcohol with meals had reduced odds of oral and pharyngeal cancer (OR = 0.08, 95% CI: 0.01–0.57), oral cavity cancer (OR = 0.07, 95% CI: 0.01–0.84), and pharyngeal cancer (OR = 0.04, 95% CI: 0.003–0.58) compared to those who do not drink with meals. For oral cavity and pharyngeal cancer, the inverse relationship ranged from MR‐Egger *β* = −18.94 ± 7.43 to MRlap *β* = −0.85 ± 0.32. Significant directional pleiotropy was confirmed (MR‐Egger intercept *p* = 0.036), but no evidence of heterogeneity (*p* > 0.05), except for MR‐RAPS heterogeneity test, which was significant (*p* = 0.035). With respect to oral cavity cancer and pharyngeal cancer tested individually, alcohol intake with meals showed a similar inverse effect with directional pleiotropy only for the former (Table S2). With respect to the number of alcoholic drinks per week, we observed a relationship with stomatitis with an IVW estimate of β = 0.60 ± 0.22 (OR = 1.83, 95% CI: 1.19–2.81), which was consistent with most of the other MR methods used (Figure 3, Table S3). No pleiotropy and heterogeneity were observed for this association (Table S3). However, the sensitivity analyses performed highlighted possible confounders in the effect of alcoholic drinks per week on the other oral health outcomes (Table S3). In the MR analyses related to maximum habitual alcohol use, none of the results survived multiple testing correction (Table S4). Because of the complex interplay among alcohol drinking behaviors, socioeconomic factors, and human health (Collins 2016; Probst et al. 2020), we conducted a multivariable MR to estimate the direct effect of alcohol drinking patterns on oral health, accounting for indirect effects related to household income. Interestingly, while the effect of alcohol intake frequency on oral and pharyngeal cancer remained significant in this multivariable analysis (Figure 4, Table S5), with a one‐category increase in frequency associated with 3.22‐fold increased odds of oral cavity cancer (OR = 3.22, 95% CI: 1.83–5.66) and 2.55‐fold increased odds of oral and pharyngeal cancer (OR = 2.55, 95% CI: 1.55–4.21) after income adjustment, the inverse relationship between alcohol intake with meals and oral and pharyngeal cancers became null when accounting for the effect of household income (*p* > 0.05, Table S6). In contrast, in the multivariable MR analysis drinks per week remained positively associated with stomatitis (*β* = +0.632 ± 0.221, *p* = 0.005), whereas household income was no longer significant (*β* = −0.499 ± 0.309, *p* = 0.110) (Table S7). No heterogeneity was observed in any of the multivariable MR analyses performed (heterogeneity *p* > 0.05; Tables S5, S6, and S7).

To further explore whether the effect of alcohol intake frequency on oropharyngeal cancer is independent of smoking and risk‐taking behaviors, we conducted additional multivariable MR analyses. The direct effect of alcohol intake frequency remained significant when modeled together with household income (*β* = 0.384, *p* = 0.039), smoking (*β* = 0.611, *p* = 0.003), and risk taking (*β* = 0.616, *p* = 0.001), as well as in a model including all four exposures simultaneously (*β* = 0.446, *p* = 0.039; Table S9). However, the conditional *F*‐statistics for the co‐exposures were low in several models, particularly for household income (*F* = 1.6), cigarettes per day (*F* = 2.2), and risk taking (*F* = 2.6), indicating weak instruments for these exposures when conditioned on alcohol frequency. These results should therefore be interpreted with caution. MR‐CAUSE analysis did not distinguish between causal and sharing models for either oral or pharyngeal cancer (*p* = 0.65) or oral cavity cancer (*p* = 0.25), and the gamma credible intervals spanned the null for both outcomes (oral & pharyngeal: −0.16 to 0.58; oral cavity: −0.04 to 0.89). These results limit causal inference and suggest that correlated pleiotropy cannot be fully excluded, although the direction of effect remained consistent with a causal interpretation across both outcomes (Table S8).

## 4 Discussion

There is an ongoing debate regarding whether the impact of alcohol intake on oropharyngeal cancer and other oral health outcomes differs depending on the amount consumed and the drinking patterns (Jun et al. 2023). The genetic instruments for alcohol intake frequency are enriched in variants related to alcohol metabolism, particularly in the ADH1B gene cluster, as well as variants influencing neurobiological pathways related to reward and addiction. These instruments are therefore likely to capture biological effects of alcohol consumption itself rather than confounding factors. The carcinogenic mechanisms through which alcohol intake frequency may increase oropharyngeal cancer risk include acetaldehyde‐mediated DNA damage, oxidative stress, and local mucosal toxicity (Baan et al. 2007). Our findings are consistent with previous Mendelian randomization studies investigating alcohol consumption and head and neck cancer. Gormley et al. (2020) used a multivariable MR approach and found independent effects of both smoking and alcohol on oral and oropharyngeal cancer risk, supporting a direct causal role of alcohol intake. More recently, (Thakral et al. 2024) highlighted the importance of HPV stratification when examining alcohol and smoking effects on head and neck cancer using MR. Our study extends this literature by examining multiple distinct alcohol drinking patterns simultaneously and explicitly modeling the interplay with socioeconomic factors, which previous studies did not address. Large‐scale GWAS of alcohol drinking behaviors highlighted consistent differences that reflect the dynamics related to sociocultural and pathological factors (Kranzler et al. 2019; Sanchez‐Roige et al. 2019). Leveraging genetic associations as instrumental variables to model differences across alcohol drinking patterns, we conducted a genetically informed analysis testing their effect on oropharyngeal cancer and other oral health outcomes.

With respect to alcohol intake frequency (ranging from never to daily or almost daily), we observed that it was associated with increased odds of both oral cavity cancer and oral and pharyngeal cancer with consistent effects across MR methods. Conversely, null or inconsistent evidence was present when pharyngeal cancer was analyzed separately. This may indicate possible cancer‐site differences in the impact of alcohol intake frequency. Multiple epidemiological studies highlighted the risk of oral and pharyngeal cancers associated with alcohol drinking (Chaturvedi et al. 2022; Jun et al. 2023), with possible differences with respect to the relationship of intensity and duration of exposure on cancer‐site risk (Di Credico et al. 2020). Because MR approaches assume a linear and homogeneous causal effect of the exposure on the outcome (Sanderson et al. 2022), the site‐specific differences of the effect of alcohol intake frequency observed in our study may reflect certain scenarios where intensity and duration tend to be stable over prolonged periods.

We observed an effect of alcoholic drinks per week (aggregated across the scales used in different studies) on stomatitis. This is in line with epidemiological studies that excessive alcohol drinking is associated with increased risk of stomatitis and other periodontal diseases (Gandhi et al. 2024; Tkacz et al. 2021) While no bias appeared to affect the relationship between alcoholic drinks per week and stomatitis, sensitivity analyses highlighted potential violations of MR assumptions (i.e., genetic instruments may be associated with the outcome independently of the exposure or through an association with a confounder) when testing the effect of alcoholic drinks per week on oropharyngeal cancers. These violations were not present when analyzing the relationship between alcohol intake frequency and oropharyngeal cancers. This suggests that alcohol drinking patterns may be linked to negative oral health outcomes through distinct mechanisms. Specifically, while genetic instruments related to alcohol intake frequency showed a direct effect on oropharyngeal cancers. Genetic instruments related to alcoholic drinks per week showed potential violations of MR assumptions when examining cancer outcomes, suggesting that the relationship between drinks per week and oropharyngeal cancer may involve pleiotropic pathways. For stomatitis, however, the multivariable MR confirmed a direct effect of alcoholic drinks per week independent of household income, while the direct effect of income was not significant, suggesting that alcohol consumption has an independent effect on stomatitis risk beyond socioeconomic factors. The different associations observed between the two alcohol‐related exposures are therefore better explained by differences in their pleiotropy patterns rather than socioeconomic mediation genetic instruments for drinks per week showed assumption violations for cancer outcomes, while those for intake frequency did not (Collins 2016). Notably, extreme heterogeneity was observed for alcohol intake frequency and lichen planus (IVW *Q*‐test *p* = 8.15 × 10^−7^), without MR‐PRESSO identifying specific outlier variants (*p* = 0.690). This unusual pattern of pervasive heterogeneity without individual outliers may reflect systematic horizontal pleiotropy, whereby multiple genetic instruments affect lichen planus through pathways independent of alcohol intake frequency. This finding should be interpreted with caution and warrants further investigation.

With respect to the interplay between alcohol drinking patterns and social determinants of health, we also observed a different pattern of associations between alcohol intake during meals and oral health outcomes. Specifically, our MR analysis suggested that alcohol intake during meals was associated with reduced odds of oral cavity cancer, pharyngeal cancer, and oropharyngeal cancer. However, our multivariable MR analysis showed that the protective effect of alcohol intake during meals on oropharyngeal cancer was null when accounting for household income, indicating that this association is explained by the socioeconomic patterning of drinking with meals (Barbería‐Latasa et al. 2022). While previous studies have suggested a potential protective effect of drinking with meals on cancer risk (Giovannucci 2025), for example, consuming alcohol with meals appeared to have a protective effect on the overall risk of head and neck cancer (Orchard and Gormley 2024). Similarly, consumption of alcohol with meals was also associated with reduced lung cancer (Chen et al. 2021). In a prospective study, drinking without meals was associated with significantly greater risks of liver cancer, liver cirrhosis, and alcoholic liver disease compared with drinking with meals (Im et al. 2021). Our findings suggest these observations may reflect socioeconomic confounding rather than a true protective biological mechanism. This strongly highlights the need to accurately model the relationship between alcohol drinking behaviors and negative health outcomes in the context of social determinants of health.

This study has several limitations. First, our study was conducted leveraging genome‐wide information generated from EUR individuals, because of the lack of large‐scale datasets informative of other population groups. This limits the generalizability of our findings to other populations, especially considering the potential differences in the interplay among alcohol drinking behaviors, social determinants of health, and negative health outcomes. Second, our analyses relied on alcohol drinking behaviors assessed using self‐reported information, which may introduce misclassification biases affecting our results (Xue et al. 2021). Third, while MR sensitivity analyses confirmed the reliability of our main findings, we cannot exclude biases related to unmeasured confounders and reverse causation. Additionally, the different patterns of associations across the alcohol drinking behaviors may have been influenced by the statistical power of the GWAS datasets. For instance, the null results observed with respect to maximum habitual intake are likely due to the limited power of the dataset investigated. Finally, household income was used as a proxy for socioeconomic status. This may have only partially captured the dimensions related to social determinants of health and their relationship with oropharyngeal cancers. Furthermore, household income shares genetic architecture with educational attainment and cognitive function, which may violate the exclusion restriction assumption of the multivariable MR if income instruments affect oral health outcomes through these correlated pathways. While heterogeneity tests did not indicate violations of instrument validity in our analyses, this limitation should be considered when interpreting the MVMR results. Finally, while reverse causation cannot be formally excluded, the use of germline genetic variants as instruments inherently protects against this bias, as genetic variants are fixed at conception and cannot be influenced by disease status. However, it should be noted that the exposure GWAS were based on self‐reported alcohol consumption, which may be subject to reporting bias if subclinical disease influenced drinking behavior prior to diagnosis. Additionally, it should be noted that MR captures a lifecourse effect of the exposure, reflecting the cumulative influence of genetically proxied alcohol consumption from birth rather than exposure at a specific time point. Additionally, the cancer outcome GWAS had limited statistical power due to small case numbers per subsite (~1000 cases in INHANCE). Our findings also lack independent replication. The INHANCE data do not distinguish between HPV‐positive and HPV‐negative oropharyngeal cancers, which are biologically distinct entities. Finally, UK Biobank is subject to healthy volunteer bias and may not adequately capture heavy drinking behaviors in high‐risk populations.

Overall, the present study provides evidence consistent with a causal effect of alcohol consumption on increased cancer risk, specifically with respect to oral and pharyngeal cancers, under Mendelian randomization assumptions. Furthermore, our findings support that the relationship between alcohol consumption and cancer risk may be differentially affected by socioeconomic factors depending on the alcohol drinking behavior investigated. In particular, alcohol intake with meals—previously considered a non‐risky alcohol‐drinking behavior—is linked to dynamics related to household income, and this can lead to inaccurate results if the latter is not properly accounted for. Additionally, the different effects observed between alcohol intake frequency and alcoholic drinks per week suggest that the scales used to assess alcohol consumption are an important aspect to consider when assessing risk of oropharyngeal cancer or other negative oral health outcomes. Taken together, our findings demonstrate that conceptualizing alcohol consumption as a homogeneous exposure is inadequate for understanding risk and guiding prevention. Alcohol use comprises diverse patterns and contexts that are strongly influenced by socioeconomic factors, which in turn shape both behaviors and health outcomes. Thus, effective cancer prevention and public health strategies must move beyond one‐size‐fits all recommendations, incorporating nuanced approaches that address both specific drinking behaviors and the underlying social determinants affecting vulnerable populations. Notably, the World Health Organization has recently concluded that there is no safe amount or frequency of alcohol consumption in terms of cancer risk, further underscoring the public health importance of our findings (WHO 2023).

## Author Contributions

T.A. contributed to project administration, conceptualization, methodology, data curation, analysis, investigation, wrote the original draft of the manuscript, and participated in writing – review and editing. R.P. contributed to supervision, conceptualization, methodology, visualization, data curation, and writing – review and editing. S.L. contributed to conceptualization, methodology, investigation, project administration, and writing – review and editing. B.C.‐M. contributed to data curation, conceptualization, visualization, and writing – review and editing.

## Conflicts of Interest

The authors declare no conflicts of interest.

## Supporting information


**Table S1:** Effect of genetically inferred alcohol intake frequency on oral health outcomes.
**Table S2:** Effect of genetically inferred alcohol intake with meals on oral health outcomes.
**Table S3:** Effect of genetically inferred alcoholic drinks per week on oral health outcomes.
**Table S4:** Effect of genetically inferred maximum habitual alcohol intake on oral health outcomes.
**Table S5:** Direct effect of alcohol intake frequency and household income on oral and pharyngeal cancers.
**Table S6:** Direct effect of alcohol intake with meals and household income on oral and pharyngeal cancers.
**Table S7:** Direct effect of alcoholic drinks per week and household income on stomatitis.
**Table S8:** MR‐CAUSE results for alcohol intake frequency and oropharyngeal cancer outcomes.
**Table S9:** Direct effect of alcohol intake frequency on oral and pharyngeal cancer across multivariable MR models including household income, smoking, and risk taking.

## Data Availability

Data used in this study are publicly available at https://gwas.mrcieu.ac.uk/datasets/ and https://console.cloud.google.com/storage/browser/finngen‐public‐data‐r12/summary_stats/. Code used in the present study will be publicly available after publication at this link: https://github.com/alahdalta1/Mendelian‐Randomization.
